# The Neural Bases of Social Intention Understanding: The Role of Interaction Goals

**DOI:** 10.1371/journal.pone.0042347

**Published:** 2012-07-27

**Authors:** Nicola Canessa, Federica Alemanno, Federica Riva, Alberto Zani, Alice Mado Proverbio, Nicola Mannara, Daniela Perani, Stefano F. Cappa

**Affiliations:** 1 Center for Cognitive Neuroscience, Vita-Salute San Raffaele University, Milan, Italy; 2 CERMAC, Vita-Salute San Raffaele University, Milan, Italy; 3 Division of Neuroscience, San Raffaele Scientific Institute, Milan, Italy; 4 Department of Psychology, University of Milano-Bicocca, Milan, Italy; 5 Institute of Molecular Bioimaging and Physiology, Consiglio Nazionale delle Ricerche, Milan-Segrate, Italy; 6 Nuclear Medicine Unit, San Raffaele Scientific Institute, Milan, Italy; Katholieke Universiteit Leuven, Belgium

## Abstract

Decoding others' intentions is a crucial aspect of social cognition. Neuroimaging studies suggest that inferring *immediate* goals engages the neural system for action understanding (i.e. mirror system), while the decoding of *long-term* intentions requires the system subserving the attribution of mental states (i.e. mentalizing). A controversial issue, stimulated by recent inconsistent results, concerns whether the two systems are concurrently vs. exclusively involved in intention understanding. This issue is particularly relevant in the case of social interactions, whose processing has been mostly, but not uncontroversially, associated with the mentalizing system. We tested the alternative hypothesis that the relative contribution of the two systems in intention understanding may also depend on the shared *goal* of interacting agents. To this purpose, 27 participants observed social interactions differing in their cooperative vs. affective shared goal during functional-Magnetic-Resonance-Imaging. The processing of both types of interactions activated the right temporo-parietal junction involved in mentalizing on action goals. Additionally, whole-brain and regions-of-interest analyses showed that the action understanding system (inferior prefrontal-parietal cortex) was more strongly activated by cooperative interactions, while the mentalizing-proper system (medial prefrontal cortex) was more strongly engaged by affective interactions. These differences were modulated by individual differences in empathizing. Both systems can thus be involved in understanding social intentions, with a relative weighting depending on the specific shared goal of the interaction.

## Introduction

How our brains make sense of other people, by inferring their *intentions* in order to be able to quickly understand and predict their behavior, is one of the main subjects of social cognitive neuroscience. A wealth of research suggests that different levels of intentionality, depending on both the sequence of execution and level of abstraction, may be associated with the involvement of specific neural systems [1], [2].

The motor *mirror system*, including premotor cortex (PMC) and inferior parietal lobule (IPL) [3]–[5], allows the automatic understanding of so-called *action goals* (e.g. the motor act of reaching/grasping a cookie) [6], [7], *immediate goals* (the goal of taking the cookie) [1], as well as “*task goals*” (or “private” intentions), such as taking a cookie in order to eat it vs. to clean the table [8]–[10]. The same private intention, however, can entail different long-term intentions, not immediately derivable from the observed scene [1], [11]. For example, one may grasp a cookie to eat it because she/he will skip dinner to complete a paper, or as a way to show appreciation of an invitation to a tea party. Due to their abstract nature and/or extended temporal scope, such long-term intentions have been associated with a *mentalizing* (Theory-of-Mind, ToM) *system*. The latter, involving the medial precuneus, temporo-parietal junction (TPJ), ventromedial (vmPFC) and dorsomedial (dmPFC) prefrontal cortex, allows to infer others' thoughts and beliefs, as well as personal traits and dispositions [12]–[15].

The simplicity of these anatomo-functional distinctions, however, conflicts with recent inconsistent results, raising two main issues.

The first concerns the *relative role* of the action understanding and mentalizing systems that, according to a recent meta-analysis [2], are usually not concurrently active and exert complementary roles in the processing of others' intentions depending on both instructions (implicit/explicit) and nature of information provided (biological/abstract; see Discussion). Yet, against this proposal a recent study showed that both systems are activated while observing point-light displays of *interacting*, compared with independent, agents [16].

The latter result is related to a second controversial issue, that concerns the brain system(s) supporting the comprehension of *social interactions*. To date, indeed, the studies investigating the neural bases of action and intention understanding have mostly employed stimuli representing single individuals, in a visual or verbal format. Many of the relevant real-life social situations, however, involve *interacting* agents and subtle cues, as those related with facial expressions and body-parts movements, which may suggest different goals despite similar overall contexts. Social interactions may thus represent a more direct test of hypotheses on intention understanding. Indeed, they are at the core of Heider and Simmel's pioneering studies in which the behavior of purely geometrical entities was interpreted in terms of cooperative or affective intentions [17]. Recent neuroimaging studies, reporting the activation of mentalizing regions while processing interactions between abstract or human agents [18]–[22], suggested that the dmPFC, the core region of the mentalizing-proper system [2], is specifically involved in understanding intentions in actual or implied social interactions. Still, this proposal conflicts with the activation of “mirror” regions, besides the mentalizing, system, in tasks involving social interactions [16], [23].

Such discrepancies suggest that both the hypothesis of a clear-cut distinction between the role of the mirror and mentalizing systems according to modality of presentation and type of instructions, and of a complete identification between processing of social interactions and involvement of the dmPFC, require further consideration. A refined hypothesis suggests that the specific *content* of the information processed may represent an additional factor responsible for the engagement of the two systems when attending social interactions.

**Figure 1:**
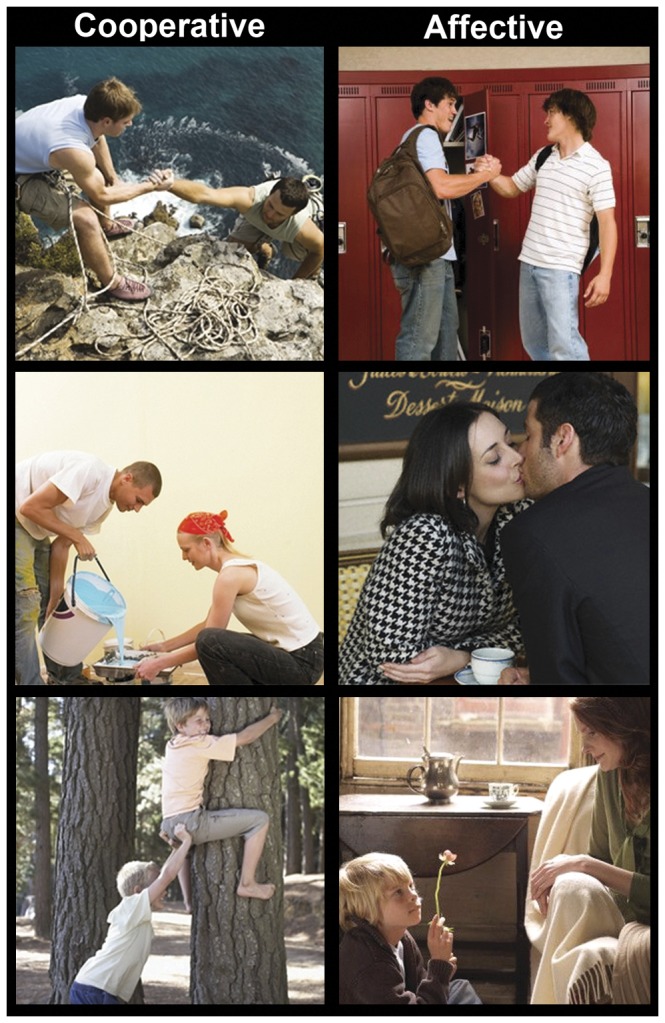
Experimental stimuli. Examples of pictures depicting cooperative (left) and affective (right) social interactions. This Figure has been previously published in PLoS ONE [54], and all the images it includes were downloaded from Google Images.

We tested this hypothesis with functional-Magnetic-Resonance-Imaging (*f*MRI) during the observation of pictures depicting either cooperative or affective interactions. This choice was first motivated by the opportunity offered by the stimuli employed, in which purposeful interactions matched for several relevant variables reflect qualitatively different shared goals such as reaching a common aim vs. establishing an affective contact (see Methods and Figure 1). Importantly, indeed, there was no significant difference across the two conditions as to the presence of an action-state or action-goal, as well as emotional activation (see Text S1). Here, “cooperative” or “affective” is the label for the different *goals* shared by two agents who, in both picture-types, were jointly and actively engaged in purposeful interactions expressing positive emotions. Moreover, the aforecited studies by Heider and Simmel [17] suggest that this dichotomy may capture a basic distinction in social perception, thus representing an ideal test for our hypothesis. Unlike several previous studies, we aimed to replicate the features of a real social interaction. Thus, we first employed *natural* social scenes, depicting human beings within ecological social contexts rather than symbolic agents. Additionally, we investigated *spontaneous* neural activity associated with attending *different* social intentions, while ensuring and assessing participants' engagement in the observation of pictures using a secondary task which did not explicitly require the attribution of mental states.

Importantly, previous reports highlighted gender differences in the engagement of the mirror system [24]–[26], in the brain response to emotional stimuli [27]–[29] and in mentalizing [30]. Moreover, a relationship has been reported between such differences and the presence of higher empathic aptitude in females than males [31], [32]. A third issue thus concerns possible interactions between observers' gender and the goal of the attended social scene, as well as a relationship with empathizing. We addressed this issue by including in the experimental sample both male and female participants, whose empathic aptitude was measured with an ad-hoc questionnaire.

We predicted that the different shared goals of the two types of interaction would modulate the neural bases of social understanding, eliciting a different engagement of the action understanding and mentalizing systems, besides a common intention understanding system likely involving the right TPJ [33]–[36]. Our hypotheses were supported by functional results, showing significant differences between the pattern of neural activity elicited by the two picture-types and only limited commonalities among them. Such common activations involved the right TPJ, supporting previous hypotheses on its role as an interface between mirror and mentalizing systems [2], [15].

## Methods

### Participants

Twenty-seven right-handed [37] healthy subjects (14 females; females mean age = 24.9 years, standard deviation (SD) = 5.08; 13 males; mean age = 26.3 years, SD = 4.2) participated in the study. All subjects had normal or corrected-to-normal visual acuity, and all reported no history of psychiatric or neurological disorders, and no current use of psychoactive medications. They gave their written informed consent to the experimental procedure, which was approved by the Ethics Committee of San Raffaele Scientific Institute.

### Stimuli, task and experimental procedure

Stimulus-set comprised 260 color pictures depicting male and female individuals of various ages actively engaged in goal-directed interactions belonging to the human repertoire and expressing positive emotions (see Figure 1). The action's goal might consist in reaching a common aim (such as raising a box, or helping each other climb a tree) (130 cooperative actions), or it might be of a purely social nature (to establish an affective contact or just to relate to someone else, as for example shaking hands, or drinking a toast to somebody) (130 affective actions). Pictures were selected so as to highlight different goals (cooperative vs. affective) of two agents who were jointly and actively engaged in purposeful interactions, yet with no significant difference across the two conditions in terms of potential confounding factors such as: the presence of an “action-state” or an “action-goal”; emotional salience; gender, age and number of persons, as well as body-parts (whole-length bodies vs. half-length bodies) and objects depicted (see Text S1 for details on stimuli selection).

In order to ensure and assess participants' engagement in the observation of pictures, we introduced a secondary task unrelated with mental state attribution. Namely, 44 further pictures depicting common natural or urban landscapes without visible persons (including streets, offices, shops, public library, countryside, seascape, mountain landscape, etc.), matched to human pictures for size, were also included. Participants were asked to carefully observe all pictures, and to press the response-key with the index finger when a landscape picture disappeared. This task was preferred over other possible tasks (e.g. to press when a cooperative action was shown) in order to avoid a conscious awareness of two types of behavior in the observed images. Indeed, a post-scanning debriefing session confirmed that no subject realized the two-fold nature of the interactions displayed.

The outer background of all pictures was dark grey, and their average luminance was 15.48 Foot-lamberts, with no significant difference across conditions as shown by Analysis of Variance (ANOVA). Pictures were shown at the centre of the screen for 1300 ms, and they were separated by a red fixation-cross whose duration was varied (“jittered”) at every trial, in order to desynchronize the timings of event-types with respect to the acquisition of single slices within functional volumes and to optimize statistical efficiency [38]. The OptSeq2 Toolbox (http://surfer.nmr.mgh.harvard.edu/optseq/) was used to estimate the optimal Inter-Stimulus-Intervals (ISIs; mean ISI = 2.064 s, range = 0.325–9.750 s). Pictures belonging to the three experimental conditions were equally subdivided in 4 *f*MRI-runs, each comprising 76 pictures randomly intermixed, whose order was counterbalanced for every subject. In order to prevent any lateralization-effect of the motor response on cerebral activity, participants responded to target pictures with the right hand in two out of the four runs, and with the left hand in the other two. The order of “left-hand” and “right-hand” runs was counterbalanced across both male and female participants.

Visual stimuli were viewed via a back-projection screen located in front of the scanner and a mirror placed on the head-coil. The software Presentation 11.0 (Neurobehavioral systems, Albany, CA, http://www.neurobs.com) was used both for stimuli presentation and subjects' answers recording. All participants underwent a training session, during which they were instructed to gaze at the centre of the screen and to avoid eye or body-movements during the scanning session. Moreover, they were asked to report their personal impressions about the task in a debriefing post-scanning session. After scanning they also completed an Italian version [39] of the Balanced-Emotional-Empathy-Scale (BEES) [40], a 30-items questionnaire measuring the individual tendency to empathize with others' emotional experiences.

### Behavioral data analysis

In order to assess behavioral performance, we first computed for every subject the percentage of correct responses (no key-press after either cooperative or affective pictures; key-press after landscape pictures) in each of the three conditions. The resulting scores were then used as a dependent variable in a Repeated Measures Analysis-Of-Variance (ANOVA) with picture-type and participant's gender as within- and between-subjects independent variables, respectively.

### *f*MRI-data acquisition

Anatomical T1-weighted and functional T2*-weighted MR images were acquired with a 3 Tesla Philips Achieva scanner (Philips Medical Systems, Best, NL), using an 8-channels Sense head coil (sense reduction factor = 2). Functional images were acquired using a T2*-weighted gradient-echo, echo-planar (EPI) pulse sequence (48 interleaved transverse slices, TR = 2600 ms, TE = 30 ms, flip-angle = 85 degrees, Field-of-View (FOV) = 192 mm×192 mm, slice-thickness = 2.6 mm, inter-slice gap = 0.2 mm, in-plane resolution = 3 mm×3 mm). Due to specific hypotheses concerning the involvement of the ventromedial prefrontal cortex in social cognition, we tilted the FOV 30° downwards with respect to the bi-commissural line to reduce susceptibility artefacts from this region. While resulting in the loss of signal from the occipital cuneus in some subjects, this procedure significantly enhanced data-acquisition from one of our primary regions of interest close to air/tissue interfaces. Each scanning sequence comprised 187 sequential volumes. Immediately after the functional scanning a high-resolution T1-weighted anatomical scan (150 slices, TR = 600 ms, TE = 20 ms, slice-thickness = 1 mm, in-plane resolution = 1 mm×1 mm) was acquired for each subject.

### *f*MRI-data pre-processing and statistical analysis

Image pre-processing and statistical analysis were performed using SPM8 (Wellcome Department of Cognitive Neurology, http://www.fil.ion.ucl.ac.uk/spm), implemented in Matlab v7.4 (Mathworks, Inc., Sherborn, MA) [41]. The first 5 volumes of each functional run were discarded to allow for T1 equilibration effects. All remaining 748 volumes from each subject were spatially realigned and unwarped [42], spatially normalized to the Montreal-Neurological-Institute (MNI) space [43] and resampled in 2×2×2 mm^3^ voxels, spatially smoothed with an 8-mm full-width half-maximum (FWHM) isotropic Gaussian kernel, and globally scaled to 100. The resulting time series across each voxel were then high-pass filtered to 1/128 Hz, and serial autocorrelations were modelled as an AR(1) process.

In the statistical analysis we focused on the regions showing significant changes in cerebral activity related to interaction goal (cooperative vs. affective) and subjects' gender, as well as to potential relations between these factors. Statistical maps were generated using a random-effect model, implemented in a 2-levels procedure [44]. At the first (single-subject) level event-related *f*MRI responses were modeled as delta “stick” functions by a design-matrix comprising the onset of cooperative, affective or landscape picture-types. Regressors modelling events were convolved with a canonical hemodynamic response function (HRF), along with its temporal and dispersion derivatives, and parameter estimates for all regressors were obtained by maximum-likelihood estimation. At the second level, random-effect group analyses across the 27 subjects were computed by means of a full-factorial design with sphericity-correction for repeated measures [45]. Several analyses were run. First, we assessed the cerebral regions recruited by the observation of either cooperative or affective, compared with landscape, pictures. Then, a conjunction-null analysis [46] across the resulting statistical maps highlighted the regions activated by the generic observation of interactions, regardless of their purpose. Additionally, we employed direct comparisons to investigate the main effect of picture-type (cooperative vs. affective) and gender (male vs. female participants). A 2×2 interaction between picture-type and gender was also assessed, to examine regions showing significant picture-type effects specific to either male or female participants.

A general statistical threshold of p<0.05 Family-Wise-Error (FWE) corrected for multiple comparisons, either at the single voxel level or based on cluster-extent, was used. A more lenient threshold of p<0.001 uncorrected (minimum cluster size = 40 voxels) was also used, for exploratory purposes, when analyzing the conjunction across picture-types, as well as their interaction with participants' gender.

The location of the activation foci was determined in the stereotaxic space of Talairach and Tournoux [47] after correcting for differences between the latter and the MNI coordinate systems by means of a nonlinear transformation (see http://www.mrc-cbu.cam.ac.uk/Imaging/Common/mnispace.shtml). Those cerebral regions for which maps are provided were also localized with reference to cytoarchitectonical probabilistic maps of the human brain, using the SPM-Anatomy toolbox v1.8 [48].

### Regions-Of-Interest (ROIs) analysis

We aimed to relate our results with specific social cognition processes, whose neural bases have been extensively investigated over the last years. Therefore, in order to endorse robust inferences on the functional role of activated regions we employed regions-of-interest (ROIs) analyses on coordinates reported in previous related studies (and thus anatomically independent from our own results).

**Figure 2:**
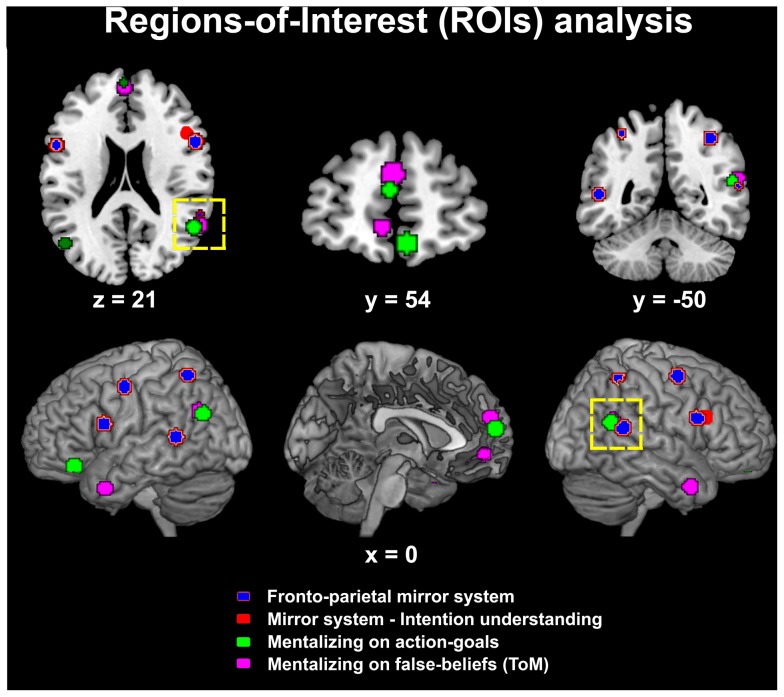
Regions-Of-Interest (ROIs) associated with social cognition. Graphical description of the regions involved in “action understanding” (i.e. mirror system; blue), “mentalizing on action goals” (green) and “Theory-of-Mind” (i.e. mentalizing proper, e.g. on false beliefs; violet) [15], along with intention understanding by the mirror system (red) [9], according to previously published papers [2], [9]. The regions were defined as 6-mm-radius spheres centred on the centre-of-mass of several previously reported MNI stereotactic coordinates for the right temporo-parietal-junction (TPJ), right dorsal and ventral premotor cortex (dPMC and vPMC), dorsomedial and ventromedial prefrontal cortex (dmPFC and vmPFC) [2] (see Methods and Tables S1, S2). A dashed yellow contour highlights the common involvement of the right TPJ in mirror motor resonance (blue), mentalizing on action goal (green) and mentalizing proper (i.e. on false beliefs, ToM; violet). The distance (in mm) from the origin of the MNI coordinate-system located in the anterior commissure is reported below each section.

In a first *explorative* step we aimed to identify the regions that, in previously published papers, have been associated with those social cognition processes which are particularly relevant for our purposes. In particular, we relied on a recent meta-analysis of over 200 studies, focusing on the involvement of several regions (STS, TPJ, ventral and dorsal PMC, dmPFC and vmPFC) in different categories of social cognition processes, ranging from perceptual analysis of biological stimuli to “mirror” resonance and mentalizing on action goals or mentalizing-proper (i.e. on false beliefs) [2]. At variance with the original meta-analysis we assessed separately the dorsal and ventral components of premotor cortex, and transformed coordinates from Talairach and Tournoux [47] to MNI space [43] with a nonlinear transformation (http://imaging.mrc-cbu.cam.ac.uk/imaging/MniTalairach). Among the reported categories we focused on regions involved in action understanding (i.e. mirror system), mentalizing on action goals, and mentalizing-proper. Since this meta-analysis did not include the temporal pole, we employed MNI coordinates reported in several studies assessing the role of this region in mentalizing (see Figure 2 and Table S1 for a description of ROIs and source-coordinates). Additionally, based on strong a priori hypotheses concerning the role of the mirror system in intention understanding, we included also the right inferior frontal gyrus MNI coordinates previously reported [9].

For all the thereby identified coordinates, we first used the SPM-toolbox Marsbar (http://marsbar.sourceforge.net) to define ROIs. To this purpose, for every combination of cognitive process, anatomical region and hemispheric lateralization (e.g. right dorsal premotor cortex in mirror activity) we first created a combined ROI comprising all the relevant MNI coordinates. The centre-of-mass of these combined ROIs was then used as the centre of 6 mm-radius spheres representing the final ROIs, that were overlaid on 3D-renders and sections of a template brain to highlight, on a purely visual basis, possible overlaps among different social cognition processes (particularly in the right TPJ; see Figure 2 and Table S1).

This first analysis was helpful to select the regions that, in a second step, were used in ROIs statistical analyses. Here, we aimed to evaluate the involvement of a subset of these regions in the understanding of cooperative and affective social interactions, as well as a potential link between the intensity of their activation and an individual empathic aptitude. To this purpose, we selected ROIs according to strong a priori hypotheses concerning the processes that appear most relevant for the understanding of social intentions. Specifically, among the aforecited ROIs we focused on five 6 mm-radius spheres related with action and intention understanding in the right dorsal and ventral premotor portion of the mirror system [9], mentalizing on action goals in the right TPJ [33]–[36] (also highlighted by the first visual step of ROIs analysis), and mentalizing-proper in the ventromedial and dorsomedial prefrontal cortex [15] (see Figure 2 and Table S2). We used the toolbox REX (http://web.mit.edu/swg) to extract from these ROIs condition-specific parameter estimates for off-line statistical analyses. In different analyses we focused on the effects of the two experimental conditions in isolation, direct comparison between them, interactions with gender and correlations with individual differences in empathizing, after testing the normal distribution of the data. We set the threshold for statistical significance at p<0.05 corrected for multiple comparisons. However, both for explorative purposes and to relate results with whole-brain neuroimaging findings, we also report ROIs surviving an uncorrected p<0.05 threshold.

This procedure allowed to assess, for the selected ROIs, condition-specific activity in coordinates that were at the same time dependent on previous literature concerning a priori identified relevant processes of interest, but still independent from results of a single study, as well as of the present study.

## Results

The behavioral assessment of participants' responses highlighted a high level of performance with no significant effect of picture-type (cooperative mean percentage of correct responses = 96.2%, SD = 0.8% ; affective mean = 95.9%, SD = 0. 8%; landscape mean = 96.7%, SD = 0. 9%; F(2,50) = 0.238, p>0.05) and participants's gender (F(1,25) = 0.000, p>0.05), nor an interaction between picture-type and participants's gender (F(2,50) = 1.347, p>0.05). These results confirmed that all picture-types were carefully observed by both male and female participants.

**Figure 3:**
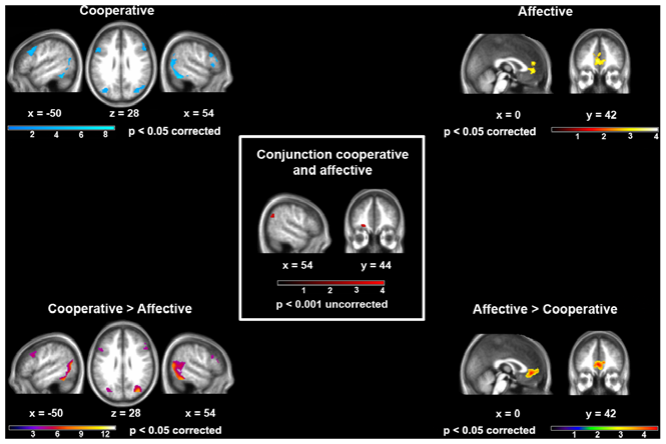
The effect of interaction goal on the neural processing of human social interactions. The brain regions activated during the observation of either cooperative (top left) or affective (top right) social interactions, as well as those more strongly activated by cooperative vs. affective (bottom left) or affective vs. cooperative (bottom right) social interactions (p<0.05 FWE corrected for multiple comparisons). The brain regions that were commonly activated while observing cooperative and affective interactions (p<0.001 uncorrected for multiple comparisons) are shown in the centre of the figure. Functional activations have been overlaid on an average-brain from individual subjects' T1-weighted images. The distance (in mm) from the origin of the MNI coordinate-system located in the anterior commissure is reported below each section.

Turning to brain activity, both the statistical maps of the single conditions, and the direct comparisons between them, highlighted different cerebral regions recruited by the processing of cooperative and affective social interactions compared with landscape pictures (see Figure 3 and Tables 1, 2, 3 and 4).

**Table 1:** Neural bases of observing cooperative social interactions.

K	H	Anatomical region (BA/region)		MNI		Voxel T-score	Cluster p-value
			x	y	z		
340	L	IFG pars Opercularis (44*)	−50	16	32	4.90	0.002
	L	Precentral Gyrus (44*)	−52	10	40	4.36	
	L	Middle Frontal Gyrus	−42	22	46	3.55	
197	R	Middle Frontal Gyrus	40	2	58	4.85	0.026
	R	Superior Frontal Gyrus	30	2	64	4.61	
	R	Precentral gyrus	46	4	42	2.98	
391	R	IFG pars Orbitalis	42	40	−14	4.92	0.002
	R	IFG pars Triangularis	52	36	18	4.92	
	R	Middle Frontal Gyrus	50	38	20	4.26	
	R	IFG pars Triangularis (45*)	54	34	14	4.14	
	R	IFG pars Opercularis (45*)	56	20	28	4.11	
	R	Middle Orbital Gyrus	40	48	−12	3.95	
177	R	IFG pars Orbitalis	36	24	−24	3.77	0.031
7955	R	Inferior Temporal Gyrus	50	−48	−26	8.73	<0.0001
	R	Fusiform Gyrus	34	−56	−18	6.83	
	L	Inferior Temporal Gyrus	−54	−56	−20	6.52	
	R	Precuneus (SPL 7P*)	10	−74	54	6.00	
	R	Middle Occipital Gyrus	40	−72	14	5.96	
	R	Middle Temporal Gyrus	50	−72	10	5.95	
	L	Precuneus	−8	−76	52	5.60	
	L	Superior parietal lobule	−22	−66	56	5.05	
	R	Superior parietal lobule	24	−70	54	4.88	
	R	Cuneus	12	−84	40	4.67	
	R	Superior occipital gyrus	30	−76	44	4.48	
	R	Inferior occipital gyrus	38	−62	−8	4.39	
	L	Fusiform gyrus	−42	−54	−24	5.46	
	L	Inferior occipital gyrus	−38	−62	−8	4.32	
	L	Middle Temporal Gyrus	−42	−64	−4	4.25	

The cerebral regions that were significantly activated during the observation of cooperative social interactions compared with landscape pictures (p<0.05 corrected for multiple comparisons).

K = cluster-extension in number of voxels (2×2×2 mm^3^), H = Hemisphere, L = Left, R = Right, BA = Brodmann area, IFG = Inferior Frontal Gyrus, SPL = Superior Parietal Lobule. An asterisk in the “Anatomical region” column denotes assignment by the Anatomy-Toolbox [48].

**Table 2:** Neural bases of observing affective social interactions.

K	H	Anatomical region (BA/region)		MNI		Voxel T-score	Cluster p-value
			x	y	z		
485	L	Mid Orbital Gyrus	−8	48	−6	3.94	<0.0001
	R	Mid Orbital Gyrus	4	46	−2	3.40	
	L	Anterior Cingulate Cortex	−8	46	−2	3.94	
	R	Anterior Cingulate Cortex	10	40	2	3.37	
	L/R	Anterior Cingulate Cortex	0	46	16	3.35	
	R	Anterior Cingulate Cortex	2	50	16	3.21	

The cerebral regions that were significantly activated during the observation of affective social interactions compared with landscape pictures (p<0.05 corrected for multiple comparisons).

K = cluster-extension in number of voxels (2×2×2 mm^3^), H = Hemisphere, L = Left, R = Right, BA = Brodmann area.

**Table 3:** Direct comparisons: cooperative vs. affective social interactions.

K	H	Anatomical region (BA/region)		MNI		Voxel T-score	Cluster p-value
			x	y	z		
126	L	Superior frontal gyrus	−28	−6	60	6.33	0.0024
	L	Precentral gyrus	−34	−2	62	3.68	
119	L	IFG pars triangularis (45*)	−48	36	16	5.26	0.0028
	L	Middle frontal gyrus	−42	50	10	4.10	
111	L	IFG pars opercularis (44*)	−48	8	24	4.64	0.0036
	L	Precentral gyrus	−40	−2	30	3.94	
128	L	Insula lobe	−34	20	−2	5.31	0.0024
	L	IFG pars orbitalis	−32	28	−4	4.16	
467	R	Precentral gyrus	42	4	30	6.04	<0.0001
	R	Middle frontal gyrus	48	30	32	3.99	
	R	IFG pars opercularis (44*)	46	8	28	5.81	
	R	IFG pars triangularis	48	24	28	4.72	
399	R	Precentral gyrus	34	−2	46	7.31	<0.0001
	R	Superior frontal gyrus	26	−2	64	4.24	
311	R	Insula lobe	38	18	6	5.32	<0.0001
	R	IFG pars opercularis (44*)	50	12	4	4.32	
	R	IFG pars triangularis	36	28	10	6.50	
630	R	Middle cingulate cortex	8	14	44	6.08	<0.0001
	R	Middle cingulate cortex	6	34	30	4.17	
	R	Superior medial gyrus	6	24	44	5.56	
12433	R	Lingual gyrus	26	−56	−10	13.25	<0.0001
	L	Fusiform gyrus	−28	−56	−12	14.32	
	R	Fusiform gyrus	32	−44	−12	9.86	
	L	Middle occipital gyrus	−28	−78	26	9.40	
	R	Middle occipital gyrus	34	−76	28	11.88	
	L	Superior occipital gyrus	−26	−80	30	9.43	
	R	Superior occipital gyrus	20	−76	40	6.74	
	L	Inferior temporal gyrus	−50	−60	−10	8.58	
	R	Inferior temporal gyrus	54	−58	−8	6.65	
	R	Angular gyrus (hIP3*)	30	−60	52	7.11	
	L	Superior parietal lobule (SPL7A*)	−24	−66	54	8.26	
	L	Superior parietal lobule	−20	−76	44	6.92	
	R	Superior parietal lobule (SPL7A*)	26	−66	54	7.29	
	L	Inferior parietal lobule	−32	−58	56	6.88	
	L	Precuneus	−8	−68	54	6.74	

The cerebral regions that were more strongly activated during the observation of cooperative than affective social interactions (p<0.05 corrected for multiple comparisons).

K = cluster-extension in number of voxels (2×2×2 mm^3^), H = Hemisphere, L = Left, R = Right, BA = Brodmann area, IFG = Inferior Frontal Gyrus, SPL = Superior Parietal Lobule. An asterisk in the “Anatomical region” column denotes assignment by the Anatomy-Toolbox [48].

**Table 4:** Direct comparisons: affective vs. cooperative social interactions.

K	H	Anatomical region (BA/region)		MNI		Voxel T-score	Cluster p-value
			x	y	z		
802	R	Medial superior frontal gyrus	10	54	32	4.12	<0.0001
	R	Mid orbital gyrus	4	58	−6	3.83	
	R	Mid orbital gyrus	8	48	−10	3.47	
	L	Medial superior frontal gyrus	−8	58	6	3.36	
	L	Medial superior frontal gyrus	−2	58	18	3.29	
	R	Medial superior frontal gyrus	14	64	4	3.28	

The cerebral regions that were more strongly activated during the observation of affective than cooperative social interactions (p<0.05 corrected for multiple comparisons).

K = cluster-extension in number of voxels (2×2×2 mm^3^), H = Hemisphere, L = Left, R = Right, BA = Brodmann area.

Observing *cooperative* scenes activated an extensive network involving the occipito-temporal cortex (occipital-face area, fusiform-face-area and extrastriate-body-area), occipito-parietal cortex, as well as inferior and superior parietal cortex (see Figure 3 and Tables 1, 3). In the frontal lobe, activations encompassed the lateral prefrontal cortex (inferior frontal, middle frontal and precentral gyri), bilaterally but with a right-hemispheric dominance. A more restricted set of areas was associated with the observation of affective social scenes, that activated the vmPFC extending into the pregenual anterior cingulate cortex (see Figure 3 and Tables 2, 4).

The evidence for common activations across the two conditions was limited. The only two foci highlighted by the conjunction-analysis, using an uncorrected p<0.001 threshold, were located in the right TPJ and left orbitofrontal cortex (see Figure 3 and Table 5).

**Table 5:** Common neural bases of observing cooperative and affective social interactions.

K	H	Anatomical region (BA/region)		MNI		Voxel T-score
			x	y	z	
40	L	Superior orbital gyrus	−24	46	−10	3.20
54	R	Angular gyrus	54	−66	30	3.16

The cerebral regions that were significantly activated during the observation of both cooperative and affective social interactions, compared with landscape pictures (p<0.001 uncorrected for multiple comparisons).

K = cluster-extension in number of voxels (2×2×2 mm^3^), H = Hemisphere, L = Left, R = Right, BA = Brodmann area.

These results were largely confirmed by ROIs analyses on functional loci previously associated with mirror and mentalizing neural activity (see Methods, Figure 2 and Tables S1, S2). Among the regions considered, only the right TPJ (associated with mentalizing on action goals) showed a trend towards significant activation (p<0.05 uncorrected) while observing *both* cooperative and affective pictures. Importantly, even at the same uncorrected threshold no significant common activations were observed in the other ROIs of the classical ToM system associated with mentalizing (e.g. on false beliefs; ventromedial or dorsomedial prefrontal cortex), nor in the mirror system.

Moreover, in line with whole-brain results activity in some ROIs was more strongly elicited by the observation of specific types of interaction (see Table S2). Stronger activations elicited by cooperative, than affective, pictures were observed in the ROIs related to the motor mirror system, namely right dorsal and ventral premotor cortex including the right inferior frontal gyrus involved in immediate/private intention understanding [9]. In contrast, stronger activations when observing affective, rather than cooperative, pictures were observed in the ventromedial prefrontal cortex previously associated with mentalizing-proper (i.e. on false beliefs) [15].

**Figure 4:**
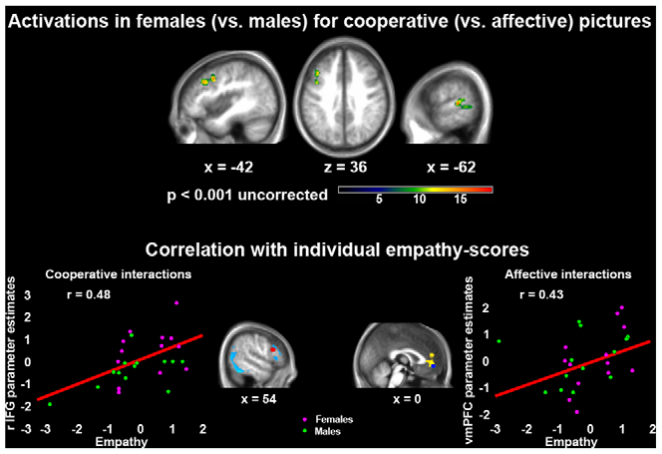
The effect of gender and empathic aptitude on the neural processing of human social interactions. Top: the brain regions that were more strongly activated in females than males when observing cooperative vs. affective interactions (2×2 interaction-analysis), overlaid on an average-brain from individual subjects' T1-weighted images (p<0.001 uncorrected for multiple comparisons). Bottom: the significant correlation between empathic aptitude and intensity of neural activity (parameter estimates) in the right IFG involved in intention understanding (red sphere) when observing cooperative interactions, and in the vmPFC involved in mentalizing (blue sphere) when observing affective interactions, in female (violet dots) and males (green dots) participants. Both parameter estimates and empathy-scores are represented as z-values in the scatterplots. The distance (in mm) from the origin of the MNI coordinate-system located in the anterior commissure is reported below each section.

When assessing whole-brain interactions between gender and picture-type, no region was more strongly activated in male than female subjects in either of the two experimental conditions, even at an uncorrected p<0.001 statistical threshold. Additionally, no region was more strongly activated in female than male subjects during the observation of affective pictures. In contrast, when observing cooperative interactions females displayed stronger activity than males in the left STS and ventral premotor cortex (see Figure 4 and Table 6). The fact that these activations encompassed the regions previously associated with action understanding was supported by the results of ROIs analyses. Indeed, stronger activations in females than males were observed, for cooperative interactions, in the ventral premotor ROI associated with intention understanding via “mirror” motor resonance (see Table S2). No gender related difference in any of the other selected ROIs was observed in the case of affective interactions.

**Table 6:** Interaction between gender and picture-type.

K	H	Anatomical region (BA/region)		MNI		Voxel T-score	Cluster p-value
			x	y	z		
224	L	Precentral gyrus	−42	8	40	4.11	0.144
	L	Middle frontal gyrus	−40	22	34	3.94	
304	L	Middle temporal gyrus	−62	−34	8	4.35	0.077
	L	Superior temporal gyrus	−56	−44	16	3.73	

The cerebral regions that were more strongly activated in females than males when observing cooperative (vs. affective) social interactions (p<0.001 uncorrected for multiple comparisons).

K = cluster-extension in number of voxels (2×2×2 mm^3^), H = Hemisphere, L = Left, R = Right, BA = Brodmann area.

Finally, within the same ROIs we examined the relationship between neural activity underpinning the observation of social interactions and individual differences in empathizing.

In line with a consistent literature [49], scores at the Balanced-Emotional-Empathy-Scale (BEES) [40] were higher for females (mean = 40.42; SD = 17.82) than males (mean = 16.46; SD = 19.87) (see Figure 4). These data are representative of the normal Italian population (female mean = 37, SD = 18; male mean = 21, SD = 18) [39], and revealed a significant gender difference (Kolmogorov-Smirnov test for normality: d = 0.14549, p>0.2; Liliefors: p>0.05; two-sample T-Test, n = 27; t(25) = 3.30; p = 0.002).

Among the considered ROIs, a significant positive correlation with empathic aptitude was observed in the right IFG, a region considered to be involved in intention understanding [9], when observing cooperative social interactions (r = 0.48; p = 0.01) (see Figure 4 and Table S2). Additionally, using an uncorrected statistical threshold we found a significant positive correlation with empathic aptitude in the medial prefrontal cortex, involved in mentalizing [15], when observing affective social interactions (r = 0.43; p = 0.027).

## Discussion

Understanding social interactions from complex visual scenes requires to infer the specific relationship occurring between the interacting agents, and their shared goal. Even more than in the case of actions performed by single individuals, this entails the processing of subtle cues that can suggest different goals. Therefore, investigating brain activity spontaneously elicited by the observation of social interactions may provide additional clues into the role of the neural mechanisms of intention understanding, as well as in the modulation of their activity by gender or by individual differences in empathizing. On these grounds, in the present *f*MRI study we addressed the brain regions involved in understanding *social* intentions, with participants observing realistic human interactions differing for their shared goal, namely reaching a common aim or establishing an affective contact, and matched for several relevant variables (see Methods and Text S1). Indeed, the absence of intrinsic differences across these interactions besides their shared goal, representing potential confounds, was confirmed by a post-scanning debriefing session, showing that no subject realized the two-fold nature of the displayed scenes.

Notwithstanding the lack of explicit awareness, however, their brain activity appeared to capture the different goals underlying them. Indeed, the differences between the pattern of neural activity elicited by the two picture-types were by far larger than the commonalities among them. Despite the presence of two interacting individuals in both scene-types, both whole-brain and ROIs direct comparisons highlighted significant activation differences across these two kinds of human interactions. Compared with affective interactions, cooperative ones elicited stronger activations in areas associated with the processing of faces, stationary bodies and biological motion (occipito-temporal and lateral temporal regions) [50], as well as in the parietal and frontal components of the motor mirror system involved in action understanding (see Figure 3 and Tables 1, 3). In particular, ROIs analyses confirmed that the right ventral premotor portion of the mirror system associated with the identification of private intentions [9] was more strongly activated by cooperative than affective interactions (see Table S2). Overall, these results suggest that the observation of cooperative interactions elicits the extraction and processing of action-related information, finally leading to the understanding of shared intentions via the mirror system. In the opposite comparison, affective interactions elicited stronger activations than cooperative ones in the medial prefrontal cortex (see Figure 3, Tables 2, 4), a core region within the mentalizing system proper that several studies have associated with the ability to make inferences about others' mental and emotional states [12], [15] (see Table S2). Both whole-brain and ROIs analyses showed that the differences between the two conditions were larger (in terms of both extent and statistical significance of the observed clusters) than the commonalities across them, which mainly involved the right TPJ and left orbitofrontal cortex (see Figure 3, Table 5 and Table S2). Importantly, it might be argued that these specific effects depend on intrinsic differences across conditions in terms of an action-state/action-goal or emotional activation, that may be more salient in cooperative or affective pictures, respectively. However, this interpretation is weakened both by stimuli selection and their evaluation by independent judges (see Methods and Text S1), as well as by the unawareness of the two-fold nature of observed interactions displayed by all subjects after scanning.

These data may contribute to the interpretation of some inconsistencies resulting from previous studies, which either suggested that the action understanding and mentalizing systems are not concurrently activated [2] or that, in contrast, both of them are recruited when observing social interactions compared with individuals acting independently from each other [16]. First, the present data support the functional segregation of the two systems, that are not concurrently activated while observing social natural scenes depicting human beings within ecological social contexts (at least with regard to the type of scenes employed here). Second, they highlight factors other than the presence of biological actions vs. abstract information, or of implicit vs. explicit instructions, as critical conditions for the engagement of the mirror or mentalizing systems, respectively [2]. The present results rather confirm the hypothesis that, besides these factors, the involvement of the two systems depends crucially on the shared *goal* of the observed social interaction. Third, at variance with some of previous studies, they show that the neural processing of social interactions is not confined to the dmPFC. It seems, instead, that observing natural social scenes of interacting individuals automatically engages, regardless of their specific goal, another portion of the mentalizing system, namely the right TPJ involved in mentalizing on action goals.

The latter region, indeed, has been consistently associated with the identification and processing of action intentionality at both the perceptual and higher-cognitive levels [15]. Such consistency, along with the intermediate anatomical localization of the TPJ in-between the STS and IPL (see Figure 2), highlighted its potential role as a bridge between mirror and mentalizing systems [2], [15]. In a reductionist attempt, it was suggested that this role may result from its involvement in the re-orientation of attention towards task-relevant directions or end-points [51]. Irrespective of the specific functional interpretation, our results support the notion of the TPJ as a low-level mentalizing region that interacts with the mirror system, and whose general role in the identification of shared goals from an observed (inter)action may represent a preliminary step for inferring more specific intentions and dispositional traits and attributes [2]. In turn, it is this latter processing that may engage, under specific conditions, the prefrontal mentalizing system proper.

Some previous results, which may appear to be at odds with this interpretation, can be explained by differences in the type of stimuli and tasks employed, as well as in the conditions across which brain activations are compared. First, as previously mentioned the mirror and mentalizing systems have been recently reported to be concurrently activated while observing point-light displays of interacting, compared with independent, agents [16]. Yet, these highly-controlled but non-ecological stimuli may have increased demands for action-processing in this study, thus highlighting the importance of realistic stimuli. On the other hand, the studies that specifically associated the processing of social interactions with the mentalizing system (dmPFC) employed tasks requiring the explicit attribution of mental states, such as choosing logical story-endings of comic-strips [22], that indeed other authors have explicitly associated with ToM processing [13], [52]. Finally, the specific involvement of the dmPFC while processing social interactions compared with single individuals [21], [53] was found in studies assessing only one type of interaction, namely relational [21] or communicative [53], thus analogous to our “affective” interactions. Their medial prefrontal activations are thus perfectly compatible with both whole-brain and ROIs results described here.

Overall, these considerations show the importance of ecological stimuli depicting realistic social interactions, in tasks that do not explicitly require social cognition processes. Additionally, they highlight the importance of tasks entailing the comparison across social interactions that differ along specific dimensions, such as their goal, to assess the degree of specificity of a given region in their general processing. The present results show that, under these conditions, a *complete* identification between the activation of ToM system and the processing of social interactions should not be taken for granted. While confirming the engagement of the medial prefrontal cortex in processing such complex stimuli [21], [22], indeed, our results show that this region is not engaged by the general processing of social interaction *per se*, but is rather preferentially activated by some of its specific contents (goals), most likely with the crucial input of the TPJ discussed above. It is worth noting that the proposed distinction between the involvement of mirror and mentalizing systems is supported, and refined in terms of time-course of activation, by recent ERP evidence obtained with an identical task and set of stimuli [54].

The involvement of mirror and mentalizing systems in processing observed social interactions highlights the important issue of *individual differences* in social cognition processes. Previous reports of gender differences related with both these systems (see Introduction) suggest possible interactions between the specific goal of the observed action (e.g. cooperative vs. affective) and gender, as well as an effect of individual differences in empathizing.

In this regard, the present results highlighted minor differences across male and female participants, which basically involved a stronger activation of the action understanding system during the observation of cooperative (vs. affective) scenes in females (vs. males) (see Figure 4, Table 6 and Table S2). Such interaction highlighted portions of the STS and ventral premotor cortex previously associated with the mirror resonance of others' actions [55]. Similar gender differences have been previously described both in the multimodal hub of biological motion perception implemented in the STS, and in regions involved in visual perception of biological stimuli (fusiform gyrus) [27], [28], [56]. Therefore, they were interpreted in terms of greater interest and attention to social stimuli in females than males. Following this view, increased attention to social stimuli in females seems to elicit increased activation of condition-specific processing mechanisms for the decoding of others' behavioral intentions, via the extraction of action-related information, when a cooperative scene is observed.

The latter interpretation was further supported by the significant positive correlation between empathy-scores (higher in females than males) and activity in the right IFG involved in intention understanding [9] during the observation of cooperative interactions (see Figure 4 and Table S2). In the case of affective interactions such a correlation involved the medial prefrontal cortex, even though only at an uncorrected statistical threshold. However, this finding is strengthened by an analogous correlation reported in a recent study employing ecological pictures of social human interactions [53]. Therefore, besides being specifically associated with the processing of different interaction goals, activity in these prefrontal regions is also positively related with an individual empathic aptitude.

Interestingly, the different neural targets of such correlations for the two types of social interaction indicate that the functional distinction between mirror and mentalizing systems for the processing of different interaction goals also reflects in a *specific* relationship with empathizing. Besides providing mutual support to each other, the two sets of results indicate that the spontaneous activation of different social cognition processes while observing interacting agents is also related to individual differences concerning empathic aptitude. Their functional segregation according to the shared goal of the observed interaction may thus provide further clues into those conditions involving abnormally low levels of empathy and deficit in spontaneous mentalizing, such as autism ([57] but see [58]), that previous studies associated with neural deficits involving either the mirror system [59] or the medial prefrontal mentalizing-proper system [60], [61].

In conclusion, the present data showed that cooperative actions aimed at a common goal preferentially activate an action understanding system for the recognition of shared intentions, by means of a direct-matching on the observer's motor representations [9], [62]. In contrast, socially-directed interactions aiming to establish an affective contact mainly engage the mentalizing system proper, centred on the medial prefrontal cortex, for processing the affective facets of observed actions. It is likely that other types of social interactions, including competitive ones, may elicit different and interesting patterns of neural activity. Future studies may address this issue by investigating further factors modulating the neural bases of social understanding, including types of stimuli (e.g. videoclips or texts vs. pictures), other types of interaction (e.g. competitive vs. cooperative) or emotional contexts (e.g. expressing negative vs. positive emotions), as well as the cognitive appraisal of the specific relationship between the two interacting agents. An exhaustive description of several types of human interaction, however, would go beyond the scope of this study, in which different shared goals emerging from social interactions represent a way to test a specific hypothesis on intention understanding.

Such an approach showed that only a specific portion of the classical ToM system, namely the TPJ involved in mentalizing on action goals, was automatically activated by the observation of social interaction *per se*, i.e. regardless of its purpose. As previously suggested [2], [15], this common activation may reflect the interaction between the two systems as a necessary step for high-level attribution of mental states (i.e. mentalizing-proper), most likely by higher-level mentalizing systems involving the medial prefrontal cortex. This hypothesis was supported by the specific content of the observed interactions leading to a differential involvement of the two systems.

Importantly, this specificity reflects a basic distinction in social cognition, between mental inferences of transitory states such as goals and intentions, and more abstract inferences of enduring characteristics, such as personality traits and stable dispositions [2], [63]. Here we show that this distinction is rooted in different brain systems, namely, the mirror and the mentalizing systems, with a potential role of the TPJ as an interface between them. Such specificity adds to other dichotomies that have been proposed to account for their differential involvement, such as input-modality, instructions, or number of agents involved, as it appears to reflect the content, or goal, of the observed interaction.

## Supporting Information

Text S1 **Description of the procedures for stimuli selection and assessment.**
(DOC)

Table S1 **Localization, coordinates and functional role in social cognition of the brain regions employed in ROIs analyses.**
(PDF)

Table S2 **Results of ROIs analyses on the brain regions associated with “mirror” motor resonance, intention understanding, mentalizing on action goals and mentalizing-proper (i.e. on false beliefs).**
(PDF)
