# Supplementary material for: The Neural Bases of Social Intention Understanding: The Role of Interaction Goals
Source: PLoS One. 2012 Jul 27;7(7):e42347. doi: 10.1371/journal.pone.0042347 (PMC3407127; doi:10.1371/journal.pone.0042347)
Supplement: Text S1 — Description of the procedures for stimuli selection and assessment. (DOC) [file pone.0042347.s001.doc]

**Text S1: stimuli selection and assessment**

The 260 pictures comprising the final set of stimuli were selected from a wider sample of 310 photos, including 155 items for each condition. For selection purposes, they were randomly presented to a group of 52 different judges of similar age and educational level as the experimental subjects (26 females; mean age = 25.7 years, SD = 3.25). Half of them judged the pictures for their cooperative content, while the other half judged them for their affective content. The risk of a position-bias in the responses was minimized by randomly changing the order in which the photographs were shown to each judge. They were shown the pictures, one by one for a few seconds, and asked to evaluate whether the action displayed seemed cooperative (or affective) to them on a 3-points scale (3 = very much cooperative (or affective); 2 = vaguely cooperative (or affective) 1 = not at all cooperative (or affective)). We used a cut-off value of 1.3, that lead to discard 25 cooperative and 25 affective pictures. At the end of this process 260 pictures (130 for each category) were selected, balanced for gender, age and number of persons, as well as body-parts (whole-length bodies vs. half-length bodies) and presence of objects. In a second phase we aimed to assess potential differences across picture-types in terms of either an “action-state” (i.e. of an actual ongoing action), action-goal (i.e. of a clear goal-directed action), or emotional salience. To this purpose, the 260 pictures comprising the final stimuli-set were further evaluated by three groups of 12 individuals each (half females in each group) of similar age and educational level as the experimental subjects (group 1 mean age = 24.6 years, SD = 2.45; group 2 mean age = 25.4 years, SD = 3.07; group 3 mean age = 24.9 years, SD = 3.61). The members of each group were shown the pictures one by one, and asked to evaluate how much they “convey a sense of action” (group 1), “convey a clear action-goal” (group 2), or “convey an emotional feeling” (group 3) on a 3-points scale (3 = very much; 2 = vaguely; 1 = not at all). For all questions, a paired t-test highlighted no significant difference between picture-types (*action-state*: mean cooperative = 2.35 (SD = 0.39), mean affective = 2.28 (SD = 0.38), t(258) = 1.47, p = 0.14; *action-goal*: mean cooperative = 2.33 (SD = 0.35), mean affective = 2.29 (SD = 0.37), t(258) = 1.64; p = 0.10; *emotional salience*: mean affective = 2.36 (SD = 0.39), mean cooperative = 2.28 (SD = 0.40), t(258) = 1.52; p = 0.13). Therefore, in line with the criteria for stimuli selection previously described, these dimensions do not appear to represent an intrinsic confound in the distinction between cooperative and affective pictures.
