# Supplementary material for: The Neural Bases of Social Intention Understanding: The Role of Interaction Goals
Source: PLoS One. 2012 Jul 27;7(7):e42347. doi: 10.1371/journal.pone.0042347 (PMC3407127; doi:10.1371/journal.pone.0042347)
Supplement: Table S1 — Localization, coordinates and functional role in social cognition of the brain regions employed in ROIs analyses. (PDF) [file pone.0042347.s002.pdf]

Human mirror system		left STS			right STS			left IPL			right IPL			left dPMC			right dPMC			left vPMC			right vPMC		
Iacoboni et al	2005																						46	20	22
Molnar-Szakacs et al	2005																			-56	10	20	52	8	20
Van der Graag et al	2007							-34	-52	49	34	-46	48										50	17	23
Grezes et al	2003	-51	-44	10	63	-36	13	-32	-44	50	30	-46	50	-42	-4	44	46	3	51						
Grezes et al	2003	-51	-44	10	67	-32	13	-34	-48	56	32	-48	56	-42	-4	43	40	2	48						
Koski et	2003				52	-30	28				39	-46	40							-51	10	18	54	12	20
Koski et	2002																			-58	8	14	57	14	12
Iacoboni et al	2001#1				57	-50	16																		
Iacoboni et al	2001#2				46	-64	8																		
Iacoboni et al	1999										37	-40	57							-50	12	12			
Buccino et al	2001							-36	-52	56										-64	12	20	60	8	24
Buccino et al	2001							-36	-40	52	40	-40	52	-56	-4	44	48	0	44						
Buccino et al	2001							-32	-64	60							40	-4	60						
Center of Mass		-52	-46	8	58	-45	15	-34	-55	56	36	-48	52	-48	-6	47	44	-2	55	-55	10	18	54	13	22

Intention understanding		Right IFG		
Iacoboni et al	2005	46	20	22
Iacoboni et al	2005	44	10	34
Iacoboni et al	2005	58	28	14
Iacoboni et al	2005	38	20	22
Iacoboni et al	2005	56	22	22
Iacoboni et al	2005	38	16	24
Center of Mass		48	19	23

Action goal		left TPJ			righ TPJ			dmPFC			vmPFC			lPFC		
Blakemore et al (adults)	2007	-42	-68	37				-9	62	8						
Blakemore et al (young)	2007	-45	-68	37	48	-60	22	0	53	17						
Vollm et al	2006	-53	-57	19	53	-60	17									
den Ouden et al	2005	-42	-68	37				-9	62	8						
Walter et al	2004#1				56	-49	14									
Walter et al	2004#2				50	-40	21				6	49	-13	-42	31	-14
Saxe & Wexler	2005	-48	-66	23	53	-51	25	0	59	8						
Center of Mass		-48	-67	26	53	-54	19	-3	59	15	6	51	-13	-42	33	-15

ToM beliefs		left TPJ			righ TPJ			dmPFC			vmPFC		
Saxe and Powell	2006	-50	-54	28	59	-55	17				-9	50	0
Ferstl and von Cramon	2002	-50	-65	25				-19	49	30			
Sommer et al	2007												
Perner et al	2006	-48	-54	30	53	-54	28	-15	54	30			
Saxe et al	2006	-45	-68	39	56	-54	19	0	56	8			
Saxe and Kanwisher	2003#1	-53	-57	22	50	-51	27						
Saxe and Kanwisher	2003#2	-48	-60	33	53	-49	19	6	56	14			
Vogeley et al	2001							6	56	26			
Vogeley et al	2001							6	56	2			
Gallagher et al	2000	-54	-66	22	60	-46	22	-10	48	12			
Wang et al (adults)	2006										-12	50	-10
Wang et al (children)	2006										2	44	-10
Grezes et al	2006				51	-47	23	4	48	20			
Grezes et al	2004				63	-42	22	14	65	19			
German et al	2004				59	-58	14	-9	57	25			
Spiers and Maguire	2006							6	54	22			
Center of Mass		-50	-64	28	57	-53	20	-1	55	23	-7	50	-4

ToM Temporal pole		left TP			right TP		
Gallagher & Frith	2004	-50	12	-24	48	20	-26
Gallagher & Frith	2004	-46	18	-26	38	6	-40
Gallagher & Frith	2004	-38	0	-42	48	8	-34
Gallagher & Frith	2004	-36	-2	-48	40	-2	-40
Gallagher et al	2000	-48	14	-36			
Gallagher et al	2000	-48	16	-38			
Calarge et al	2004	-44	0	-28			
Castelli et al	2000	-38	-4	-32			
Fletcher et al	1995	-42	22	-20			
Heekeren et al	2003	-49	18	-20			
Schulte-ruther et al	2007	-48	12	-36	46	16	-36
Takahashi et al	2008	-50	20	-24			
Moriguchi et al	2006				50	9	-24
Moriguchi et al	2006				40	4	-34
Wakusawa et al	2007				52	12	-32
Walter et al	2004				54	0	-21
Center of Mass		-45	9	-32	47	8	-31
