# Supplementary material for: The Neural Bases of Social Intention Understanding: The Role of Interaction Goals
Source: PLoS One. 2012 Jul 27;7(7):e42347. doi: 10.1371/journal.pone.0042347 (PMC3407127; doi:10.1371/journal.pone.0042347)
Supplement: Table S2 — Results of ROIs analyses on the brain regions associated with “mirror” motor resonance, intention understanding, mentalizing on action goals and mentalizing-proper (i.e. on false beliefs). (PDF) [file pone.0042347.s003.pdf]

Process/System	Region	Side	Cooperative pictures		Affective pictures		Direct comparisons		Interaction gender-picture	Significant correlations with empathy	
			mean C	p-value	mean A	p-value	mean C vs A	p-value	p-value	condition C	condition A
mirror system	dPMC	R	0.649	0.008**			1.841	0.000**			
mirror system-intention understanding	vPMC	R	0.487	0.038*			1.316	0.001**	0.023*	0.48**	
mentalizing on action-goal	TPJ	R	0.459	0.046*	0.434	0.049*	0.25	0.23			
ToM	vmPFC				0.665	0.007**	-1.188	0.001**			0.43*
ToM	dmPFC				0.38	0.16	-0.35	0.23			
More activated in C than A; corrected for multiple comparisons											
More activated in A than C; corrected for multiple comparisons											
Common activations across C and A; uncorrected for multiple comparisons											

Legend
C = cooperative interaction
A = affective interaction
dPMC = dorsal premotor cortex
vPMC = ventral premotor cortex
TPJ = temporo-parietal junction
vmPFC = ventromedial prefrontal cortex
TP = temporal pole

The brain regions that were more strongly activated by cooperative than affective pictures (light-blue), more strongly activated by affective than cooperative pictures (blue), or commonly activated by the two interaction-types (yellow; uncorrected statistics) are reported.
For each region, the mean and statistical significance of parameter estimates in the relevant condition, as well as in direct-comparisons across conditions, are reported.
The correlation-coefficient is also reported, in the right-most part of the figure, for the two regions that showed a significant correlation with empathy scores while observing cooperative (ligh-blue; right inferior frontal gyrus involved in intention understanding) and affective (blue; ventromedial prefrontal cortex involved in mentalizing-proper; uncorrected statistics).
Double and single asterisks denote a p < 0.05 statistical threshold corrected or uncorrected for mutiple comparisons, respectively.
